# Aberrant Expression and Subcellular Localization of PER2 Promote the Progression of Oral Squamous Cell Carcinoma

**DOI:** 10.1155/2020/8587458

**Published:** 2020-02-22

**Authors:** Fengyuan Guo, Qingming Tang, Guangjin Chen, Jiwei Sun, Junyi Zhu, Yulin Jia, Wenfeng Zhang

**Affiliations:** ^1^Department of Oral and Maxillofacial Head Neck Oncology, School and Hospital of Stomatology, Wuhan University, Wuhan 430079, China; ^2^Department of Stomatology, Union Hospital, Tongji Medical College, Huazhong University of Science and Technology, Wuhan 430022, China

## Abstract

Oral squamous cell carcinoma, one of the most prevalent cancer types in the world, has been confirmed under the influence of a key circadian gene, PER2, whose role has been identified in the development of some other types of cancers. However, the mechanism through which PER2 regulates the progress of OSCC remains largely unknown. In this study, we showed that besides the abnormal expression and subcellular localization of PER2 observed in OSCC tissues and cells as expected, these anomalous changes also existed in the adjacent noncancerous tissues, which was a novel finding in our research. The phase of PER2 rhythmic expression pattern in OSCC cells was later than that in oral keratinocytes in the protein level. In addition, we demonstrated that PER2 played as a resistant factor in the development of OSCC by upregulating *TP53* and inhibiting epithelial-mesenchymal transition *in vitro* and *in vivo*. Taken together, our results identified that the development of OSCC is closely associated with PER2, the aberrant expression and subcellular localization of which facilitates the malignant progress.

## 1. Introduction

Oral squamous cell carcinoma (OSCC) is one of the most common tumors in head and neck malignancies with high cancer-related mortality rates worldwide [1]. Besides the advancing treatment, the prognosis of OSCC has not been improved substantially over the decades [2]. Emerging evidences showed that night-shift workers are at an increasing risk of developing various types of cancers, including OSCC [3–5]. However, the mechanisms underlying circadian clock functional involvements in OSCC remain largely unknown.

In mammals, the circadian clock plays a pivotal role in orchestrating physiological, biochemical, and behavior processes to better adapt to the diurnal stimulus in the environment [6, 7]. The current molecular model of circadian clock is a classical key feedback loop, where the heterodimer of CLOCK (clock circadian regulator) and BMAL1 (aryl hydrocarbon receptor nuclear translocator-like) determines the rhythmic expression of genes containing E-box elements such as PERs (periods) and *CRYs* (cryptochromes), which in turn form negative regulating complexes that interact with CLOCK-BMAL1 [6, 8, 9]. PER2 is a large and multifaceted protein with an incompletely defined domain architecture, whose functional involvement extends beyond its role as a circadian regulator [10, 11]. Previous reports showed that PER2 influences cell division, growth, and death in various human cultured cells [12, 13]. These functions were particularly due to its stability and transcriptional activity of classical tumor-related genes in response to genotoxic stress [10]. Loss of PER2 predisposes genetically engineered mice to spontaneous lymphomagenesis and decreases their susceptibility to radiation [13]. The above findings are consistent with the role of PER2 in suppressing the cancer-prone phenotype, whereas a note of caution is in order, as inconsistencies among *in vitro* and *in vivo* studies have been reported [14, 15]. Also, it has been reported that the downregulation of PER2 is observed in OSCC tissues and cells, which contributes to carcinogenesis and development of OSCC [16–18]. However, how PER2 is involved in the progress of OSCC remains largely unknown.

In this study, we found that there were significant differences between the expression and subcellular localization of PER2 in OSCC tissues and cells as well as adjacent noncancerous tissues comparing with normal oral epithelial tissues and cells. The knockdown of PER2 facilitated OSCC cell proliferation, migration, and invasion, while PER2 overexpression inhibited these processes. Next, we identified *TP53* and classical EMT (epithelial-mesenchymal transition) genes as targeted downstream genes of PER2. Our findings demonstrated that PER2, which is obviously downregulated and has an obstacle entering the nucleus in OSCC, gets involved in the processes of OSCC cell proliferation, migration, and invasion.

## 2. Materials and Methods

### 2.1. Human Specimens

A total of 36 paired human OSCC tissues and corresponding adjacent noncancerous tissues, originated from pathologically diagnosed OSCC patients without preoperative chemotherapy and radiotherapy, were obtained from the Union Hospital of Tongji Medical College, Huazhong University of Science and Technology (Wuhan, China), between June 2016 and December 2018. All tissue specimens were obtained between 10 : 00 am and 12 : 00 am. 36 cases of normal oral tissues were obtained from noncancerous patients who underwent operative resection during the same period. All patients signed an appropriate consent form for biobanking. The protocol of the study was approved by the Institutional Research Ethics Committee of Tongji Medical College (Wuhan, China). Clinical pathologic characteristics including age and sex have no differences in two groups of patients (Table 1). Samples collected were further used in immunofluorescent staining, qRT-PCR, and western blotting for detection of PER2 expression level.

### 2.2. Tissue Microarray

PER2 expression was firstly analyzed by immunohistochemistry on a tissue microarray containing 80 samples of oral cancer tissues and control tissues, including OSCC, nonsquamous cell carcinoma, and parotid gland tumor, which was purchased from Shanghai Outdo Biotech Company (Shanghai, China). Among them, there were only 10 pairs of OSCC tissues and corresponding adjacent noncancerous tissues in the tissue microarray, which were chosen and shown for our experiments.

### 2.3. Cell Culture

Three human OSCC cell lines (SCC15, SCC25, and CAL27) obtained from ATCC were used for experiments. All cells were authenticated semiannually by short tandem repeat DNA fingerprinting in China Center for Type Culture Collection (Wuhan, China).

### 2.4. Isolation, Culture, and Identification of Keratinocytes

Human oral epithelial tissues were obtained from oral noncancerous disease patients. The muscles or connective tissues were cleaned and minced using sterile microsurgical instruments and then treated with 4 mg/mL dispase II (Gibco) overnight at 4°C to separate epidermis. Trypsin-EDTA (Invitrogen) digested the epidermal layer for 20 minutes to isolate oral keratinocytes (OKCs). Then, cells were cultured at 37°C in 5.0% CO_2_, with medium (Promocell), after which the KCs were authenticated by immunofluorescence assay with rabbit antibodies of anti-cytokeratin 19 (Abcam) and anti-vimentin (Abcam) as previously described [19].

### 2.5. Immunohistochemistry

Primary antibodies for Ki67 (1 : 200, Abcam) were applied to the sections overnight in a humidified chamber at 4°C. As a next step, the slides were washed several times with PBS and then incubated with appropriate secondary antibody. The images were captured using a Nikon microscope (Japan).

### 2.6. Immunofluorescence

Cells were fixed on creep plates and incubated with the primary antibody specific for PER2 (1 : 200, Abcam) overnight at 4°C. Cells on creep plates were incubated with Alexa Fluor 488 secondary antibody (1 : 500, Abcam). Images were acquired with a laser scanning confocal microscope (Nikon, Japan) after counterstained with 4′,6-diamidino-2-phenylindole (DAPI, Abcam).

### 2.7. Nuclear Protein and Cytoplasmic Protein Extraction

Extraction of nuclear protein and cytoplasmic protein was conducted using NE-PER™ Nuclear and Cytoplasmic Extraction Reagents (ThermoFisher Scientific) following the manufacturer's protocol.

### 2.8. Plasmids Construction and Transfection

Guide RNAs targeting for the human PER2 gene were designed using Optimized Crispr Design (http://crispr.mit.edu/). To upregulate PER2 in SCC15, SCC25, and CAL27 cells, the oligos were synthesized and cloned into a pcDNA3.1-Myc-His (+) vector (Genechem, China); an empty vector was used as a negative control. To generate stably transfected cell lines, puromycin (ThermoFisher Scientific) was used to select stably transduced cells.

### 2.9. Western Blot Analysis

Cells were lysed by RIPA containing protease inhibitors (Beyotime, China). Cell extracts, standardized for protein content, were subjected to 10% SDS-PAGE for separation and blotted onto PVDF membranes (Millipore). After blocking with 5% nonfat milk, the membrane was probed with primary antibodies against PER2 (1 : 1000, Abcam), GAPDH (1 : 5000, Thermo Scientific), and H3 (1 : 5000, Abcam), respectively. Computational densitometry was quantified using ImageJ software (National Institutes of Health, USA).

### 2.10. RNA Isolation and Quantitative Real-Time PCR

Total RNA was isolated from OSCC cell lines with the TRIzol® Reagent (Vazyme, China). The cDNA was synthesized using the RevertAidTM First Strand cDNA Synthesis Kit (Takara, Japan). Quantitative analysis was performed with the SYBR Greenmaster mix (Vazyme, China). The following specific primers were used: PER2, 5′-TTCACCACATCCTGGAACAAAC-3′ (forward) and 5′-TTCACCACATCCTGGA ACAAAC-3′ (reverse); *TP53*, 5′-ATTTGATGCTGTCCCCGGACGATATTGAAC-3′ (forward) and 5′-ACCCTTTTT GGACTTCAGGTGGCTGGAGT-3′ (reverse); *MMP1*, 5′-AACATCACTTCTCCCCGAAT-3′ (forward) and 5′-GTTCCCAAAATCCTGTCC A-3′ (reverse); *ZEB1*, 5′-TGCTGTCATTTACCCCGAAG-3′ (forward) and 5′-AACT GGGAGAATGCATCTGG-3′ (reverse); *ZEB2*, 5′-AATTGTGATCCTCCGCTCAG-3′ (forward) and 5′-TCCTCGAGTGGTCCAATTTC-3′ (reverse); *TWIST1*, 5′-GGATAA GCTGAGCAAGATCCAG-3′ (forward) and 5′-CAGCTTGCCATCTTGGAGTC-3′ (reverse); *TWIST2*, 5′-GGAGTCCGCAGTCTTACGAG-3′ (forward) and 5′-TCTGGA GGACCTGGTAGAGG-3′ (reverse); *GAPDH*, 5′-ACTTTGGTATCGTGGAAGGACTCAT-3′ (forward) and 5′-GTTTTTCTAGACGGCAGGTCAGG-3′ (reverse). *GAPDH* was used as the internal control. The fold change was calculated using the 2^−ΔΔCT^ method, and all analyses are based on experiments performed in triplicate.

### 2.11. Cell Proliferation and Colony Formation Assays

Cell proliferation was detected using CCK-8 kit (Dojindo, Kumamoto, Japan) according to the instruction. Briefly, cells (2 × 10^3^ cells/well) were seeded in 96-well plates (NEST, China) and cultured for 48 h, stained with 10 *μ*L CCK-8 dye for 4 hr at 37°C. Finally, the absorbance was measured at 450 nm. For the colony formation assay, cells were seeded into 6-well plates and then cultured for 2 weeks at 37°C. Cells were fixed with formaldehyde, washed with PBS, and stained with crystal violet dye (Beyotime, China).

### 2.12. Scratch Wound-Healing Assay

Cells were grown to 90% confluence in a 12-well plate, then scratched with a sterile micropipette tip (200 *μ*L), and subsequently washed three times with PBS. Cells were cultured with medium supplemented with 1% FBS at 37°C in a 5% CO_2_ incubator. The migration distance was photographed with 6 hours of the interval from 0 hr to 48 hr to assess cell migration and finally measured using ImageJ software (National Institute for Health, USA).

### 2.13. Matrigel Invasion Assay

An invasion experiment was performed using the transwell chambers consisting of 8 *μ*m pore size membranes coated with 50 *μ*L matrigel matrix (BD Sciences). The cells were starved in serum-free medium for 24 hr and then (1 × 10^5^ cells/well) loaded in the upper chamber with media containing 0.5% FBS while medium supplemented with 10% FBS was added to the lower chamber. The cells were allowed to invade for 48 hr at 37°C in a 5% CO_2_ incubator. Then, the cells were fixed with 3.7% formaldehyde and stained with 0.5% crystal violet dye.

### 2.14. Subcutaneous Xenograft Model

A total of 36 male BALB/c nude mice (4-week-old) were purchased from the Beijing HFK Bioscience Co. Ltd to build the subcutaneous xenograft tumor model. All animal experiments were approved by the Institutional Animal Care and Use Committee of Tongji Medical College. The nude mice were randomly divided into SCC15/mock, SCC15/PER2 (PER2 overexpression), SCC15/ PER2-KD (PER2 knockdown), CAL27/mock, CAL27/PER2 (PER2 overexpression), and CAL27/PER2-KD (PER2 knockdown) groups (*n* = 6 per group). 1 × 10^7^ tumor cells suspension cultures were injected into nude mice subcutaneously. The mice were sacrificed on day 35 to compare tumor weight and volume.

### 2.15. Lung Metastasis Model

A total of 120 male BALB/c nude mice (4-week-old) were purchased to build the lung metastasis model. The experiment was shown with 6 groups (SCC15/mock, SCC15/Per2, SCC15/Per2-KD (Per2 knockdown), CAL27/mock, CAL27/Per2, and CAL27/Per2-KD). Tumor cells (3 × 10^6^) were injected into the tail vein of the nude mice (*n* = 20 per group). After 6 weeks, one-half of mice were sacrificed and the lung tissues were collected and paraffin-embedded. The specimens were sectioned for H&E staining and macroscopically detected. The other half of the mice were followed up to death.

### 2.16. Statistical Analysis

Unless otherwise stated, all data are shown as mean ± standard deviation. The GraphPad Prism 7.0 software was used for statistical analysis. Statistically significant differences between groups were determined by Student's *t*-test or ANOVA, and statistical significance was defined as *P* < 0.05.

## 3. Results

### 3.1. The Expression and Subcellular Localization of PER2 Is Noticeably Changed in OSCC Tissues and Adjacent Noncancerous Tissues

**Figure 1:**
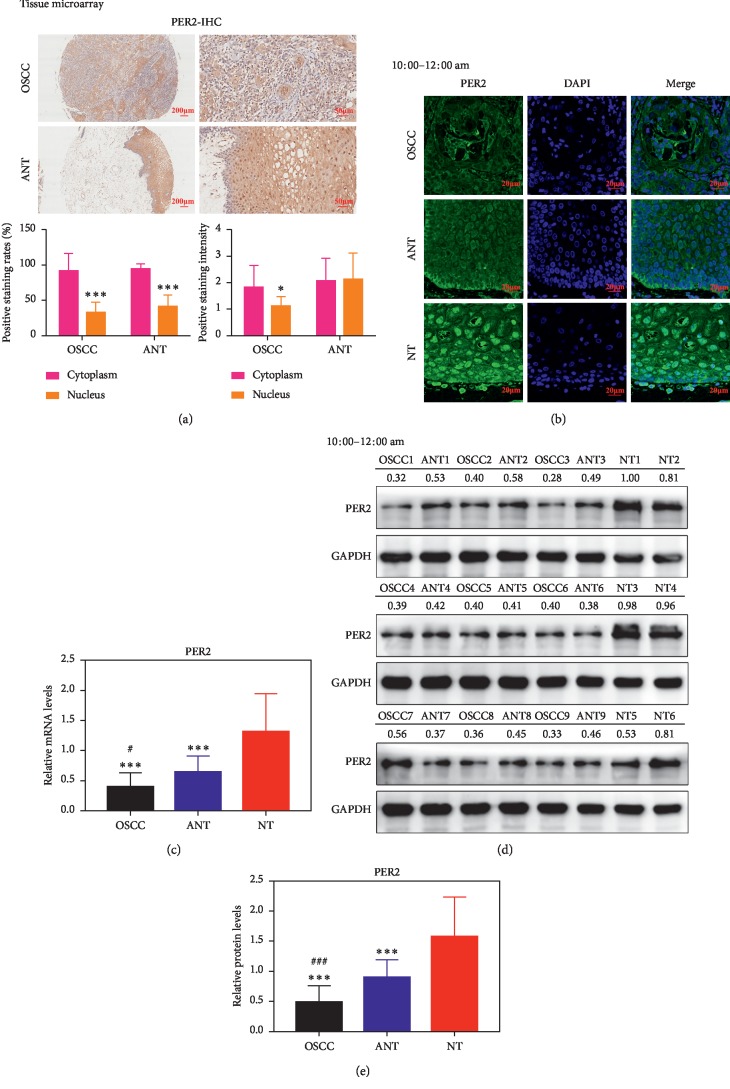
PER2 expression and nuclear translocation are disturbed in OSCC tissues and adjacent noncancerous tissues. (a) Detection of PER2 expression levels on a tissue microarray. (b) Immunofluorescence staining for PER2 (green) in the 36 paired collected tissue samples and the 36 normal samples. Nuclei were stained by DAPI (blue). (c) The relative mRNA levels of PER2 in the 36 paired collected tissue samples and the 36 normal samples. (d and e) Representative western blotting analysis of PER2 in the 36 paired collected tissue samples and the 36 normal samples. GAPDH was used as the loading control (OSCC, oral squamous cell carcinoma tissues; ANT, adjacent noncancerous tissues; NT, normal oral tissues). ^*∗∗∗*^*P* < 0.001 (compared with NT), ^#^*P* < 0.05, and ^###^*P* < 0.001 (compared with ANT).

To determine the differences of PER2 between human OSCC tissues and adjacent noncancerous tissues (ANT), immunohistochemical staining was performed on a tissue microarray where there were 10 pairs of OSCC tissues and corresponding ANT. The results revealed that cytoplasmic PER2-positive staining rates were significantly higher than that of the nucleus in both OSCC and ANT. However, the cytoplasmic PER2-positive staining intensity was higher than that of the nucleus in OSCC, whereas there was no difference in ANT (Figure 1(a)). Furthermore, confocal images of immunofluorescent indicated that PER2 expression levels were highest in normal oral tissues (NT) obtained from noncancerous patients and lowest in OSCC. Accordingly, the highest nucleic PER2 levels were in NT compared with the lowest in OSCC, suggesting the loss of PER2 nucleus-translocation function in cancer as well as in ANT (Figure 1(b)). The PER2 expression levels were determined by qRT-PCR and western blotting analysis in OSCC and paired ANT as well as NT from other patients. Results showed that the PER2 protein levels were significantly reduced in OSCC and ANT compared with those in normal tissues (Figures 1(c)–1(e)). Collectively, these results suggested that deficiency of PER2 in both expression and subcellular localization might be used as a novel precancerous prognostic marker.

### 3.2. PER2 Expression Is Remarkably Downregulated and Its Nuclear Translocation Is Blocked in OSCC Cells

**Figure 2:**
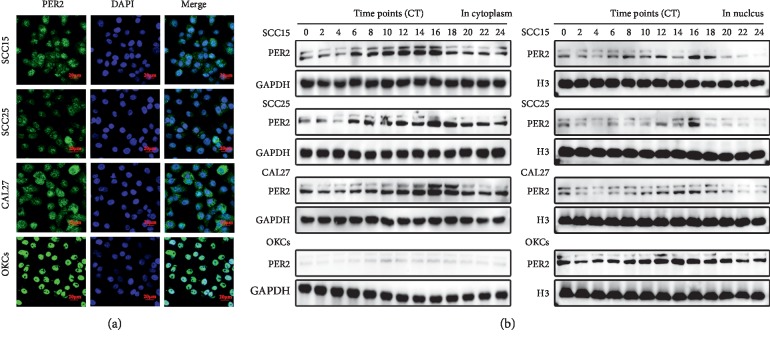
PER2 expression is remarkably downregulated and its nuclear translocation is blocked in OSCC cells. (a) Immunofluorescence staining for PER2 (green) in three OSCC cell lines and OKC cells. Nuclei were stained by DAPI (blue). (b) The PER2 protein expression levels of three OSCC cell lines in both cytoplasm and nucleus at each time point.

To further investigate the abnormal change of PER2 expression patterns of in OSCC, we cultured three classical OSCC cell lines (SCC15, SCC25, and CAL27) and used normal oral keratinocytes (OKCs) as control. As expected, immunofluorescent experiments showed that the fluorescent intensity of PER2 was lower and cytoplasmic localization of PER2 was more noticeable in OSCC cells (Figure 2(a)). Accordingly, nuclear protein and cytoplasmic protein extracts from synchronized OSCC cells and OKCs obtained at indicated time points were monitored for the rhythmic expression of PER2. Indeed, PER2 underwent a similar 24-hour oscillation in OSCC cells and OKCs, albeit with a 2 hr delay of PER2 expression in OSCC cells compared with OKCs. Similarly, the nucleic extracts from OSCC cells showed comparatively lower PER2 levels, whereas the cytoplasmic extracts from OSCC cells showed higher ones relative to OKCs (Figure 2(b)). Together, these findings showed the aberrant features of PER2 expression in OSCC cells.

### 3.3. Loss of PER2 Promotes Proliferation, Migration, and Invasion of OSCC Cells

**Figure 3:**
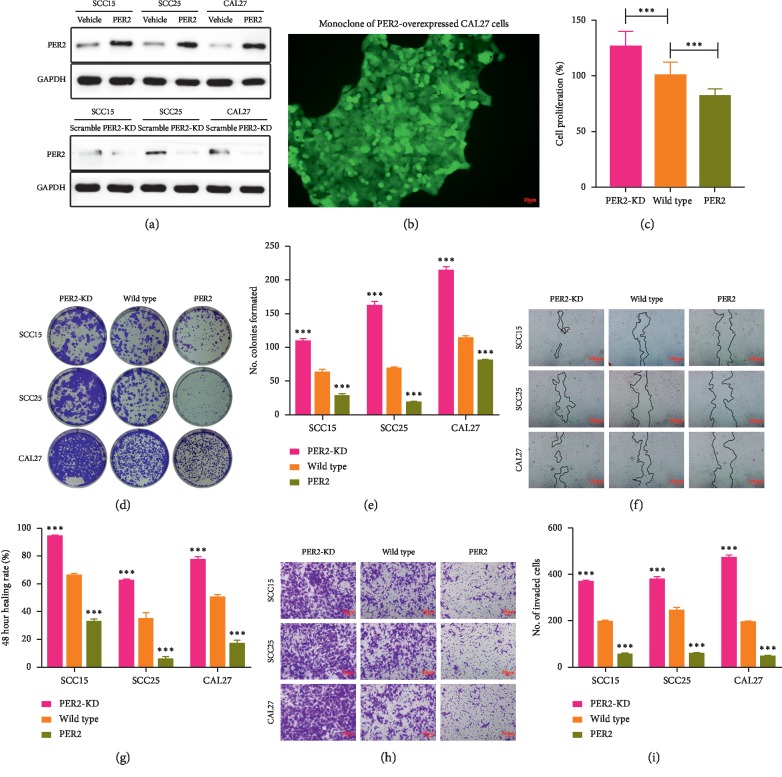
PER2 inhibits OSCC cell proliferation, migration, and invasion *in vitro*. (a) Western blotting analysis of PER2 expression. (b) The monoclone of PER2-overexpressed CAL27 cells. (c) PER2 inhibited cell proliferation of CAL27 cells. (d and e) Growth of OSCC cells was detected using the clone formation assay. (f and g) Representative microphotographs of wound-healing assays. Black lines mark the migration front. (h and i) Representative microphotographs of invasion assays.

**Figure 4:**
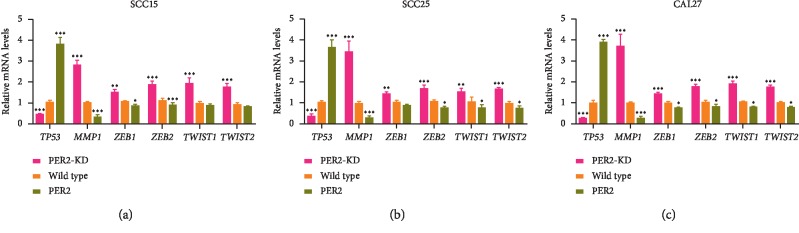
PER2 controls the EMT process in OSCC. (a-c) Quantitative RT-PCR analysis of EMT-associated genes in SCC15 (a), SCC25 (b), and CAL27 (c). ^*∗*^*P* < 0.05, ^*∗∗*^*P* < 0.01, and ^*∗∗∗*^*P* < 0.001.

To evaluate the potential role of PER2 in OSCC cells, PER2-knockdown and PER2-overexpressed cells were established. The knockdown and overexpression of PER2 were assayed using western blotting to confirm that the efficiency of gene operation was enough for our further experiments (Figure 3(a)). Overexpression of PER2 could enhance the absolute amount of PER2 in the nucleus, whereas there is no change in the ratio of nuclear/cytoplasmic PER2 expression in PER2-overexpressed OSCC cells (Figure 3(b)). The proliferative ability was highest in PER2-knockdown CAL27 cells and lowest in PER2-overexpressed CAL27 cells (Figure 3(c)). Compared with control cells, the cell colony assay manifested that the number of colonies formed by PER2-knockdown OSCC cells was increased while the number of colonies formed by PER2-overexpressed OSCC cells was decreased (Figures 3(d) and 3(e)). The wound-healing assay revealed that after 24 h of culture, the distance of migration in PER2-knockdown OSCC cells was significantly increased whereas the distance in PER2-overexpressed OSCC cells was remarkably decreased in contrast to control cells (Figures 3(f) and 3(g)). In addition, the matrigel invasion assay showed that after 48 h of culture, loss of PER2 served to enhance the ability of invasion in OSCC cells (Figures 3(h) and 3(i)). QRT-PCR was then performed on the above OSCC cells to detect the expression levels of tumor-associated genes, including *TP53* (tumor protein p53), *MMP1* (matrix metallopeptidase 1), *ZEB1/2* (zinc finger E-box binding homeobox 1/2), and *TWIST1/2* (twist family bHLH transcription factor 1/2), based on our previous RNA-sequencing results in PER2-overexpressed OSCC cells. Results revealed that knockdown of PER2 led to a decrease in expression of *TP53* as well as an increase in expression of EMT-associated genes *MMP1*, *ZEB1*, *ZEB2*, *TWIST1,* and *TWIST2* in OSCC cells (Figures 4(a)–4(c)). Taken together, we concluded that PER2 plays a negative role in cell proliferation, migration, and invasion of OSCC.

### 3.4. PER2 Overexpression Suppresses Tumor Growth in Mouse Xenograft Models

**Figure 5:**
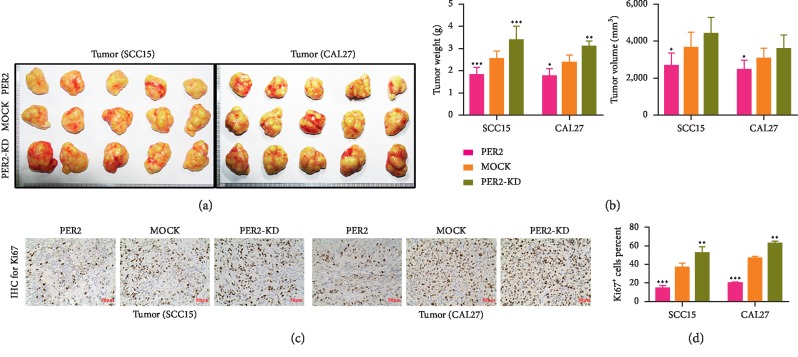
Loss of PER2 contributes to the growth of OSCC cells *in vivo*. (a and b) PER2 suppressed tumor growth in xenograft models. Representative images of tumors formed (a) and tumor weight and tumor volume curves (b) are presented. (c and d) Proliferation rates were measured using immunohistochemical staining for Ki67. Representative image of IHC staining (c) and statistical analysis (d). ^*∗*^*P* < 0.05, ^*∗∗*^*P* < 0.01, and ^*∗∗∗*^*P* < 0.001.

To determine whether PER2 has the tumor-suppressive effect *in vivo*, PER2-knockdown, PER2-overexpressed, and mock (with a GFP) SCC15 and CAL27 cells were subcutaneously implanted into the nude mice. As a result, xenografts derived from PER2-knockdown SCC15 and CAL27 cells grew noticeably faster than those coming from the mock. However, the xenografts derived from PER2-overexpressed cells were much smaller than those coming from mock cells (Figures 5(a) and 5(b)). Furthermore, immunohistochemical staining for Ki67 was carried out to see whether PER2 could influence the ability of proliferation *in vivo*. As expected, xenografts coming from PER2-knockdown OSCC cells exhibited higher Ki67-positive rates, while xenografts coming from PER2-overexpressed OSCC cells showed lower Ki67-positive rates compared with those from mock OSCC cells (Figures 5(c) and 5(d)). In summary, it was evident that PER2 took an important part in tumor suppression *in vivo*.

### 3.5. PER2 Overexpression Suppresses Tumor Metastasis of OSCC Cells

**Figure 6:**
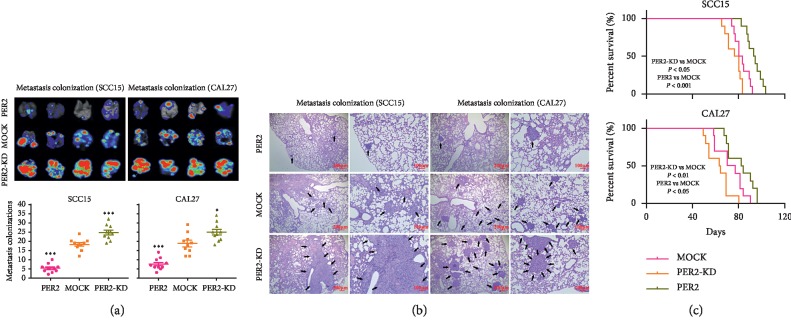
PER2 inhibits tumor metastasis and improves the prognosis of OSCC cells. (a and b) Cells were transported into mice with an injection of caudal veins and lung tissues were obtained later. Metastasis colonization of lung tissues from OSCC cells was detected by *in vivo* fluorescence imaging (a) and H&E staining (b). (c) The prognosis of metastasis models was depicted by the Kaplan–Meier curves. ^*∗*^*P* < 0.05, ^*∗∗∗*^*P* < 0.001.

Based on the findings that PER2 promoted carcinogenesis *in vivo*, further investigations were performed to explore whether PER2 could promote metastasis of OSCC cells. GFP-marked OSCC cells were injected into the blood of null mice. Immunofluorescent images of lung tissues from the mice indicated an increase of metastatic focuses derived from PER2-knockdown OSCC cells and a decrease of metastatic focuses derived from PER2-overexpressed OSCC cells, and the hematoxylin and eosin (H&E) staining results identified more metastasis from PER2-knockdown OSCC cells and less metastasis from PER2-overexpressed OSCC cells (Figures 6(a) and 6(b). Finally, Kaplan–Meier curves and log-rank tests demonstrated that the mice with the injection of PER2-knockdown OSCC cells had a shorter overall survival time, while the mice injected with PER2-overexpressed OSCC cells experienced a longer survival time in comparison to those injected with mock OSCC cells (Figure 6(c)). In conclusion, PER2 can serve as a resistance for tumor metastasis in the mouse model.

## 4. Discussion

To explore the role of the classical circadian gene PER2 in OSCC, we performed this study and revealed that PER2 expression and nucleus-translocation were both downregulated in OSCC tissues and cells as well as ANT. Similar to the results in non-small-cell lung cancer [20], PER2 overexpression significantly inhibited the proliferation, migration, and invasion of OSCC cells, indicating that PER2 might act as a crucial antitumor gene. The growth of *in vivo* tumors was also suppressed resulting from the upregulation of PER2 in derived OSCC cells.

PER2 level is significantly decreased in OSCC tissues compared with ANT, which is consistent with the previous studies that PER2 expression levels were decreased significantly in OSCC [16–18]. Most studies showed the resistant effect of PER2 in carcinogenesis, which might be similar in various types of cancers [19]. However, some previous studies also indicated that activation of PER2 can induce an increase in tumor incidence [21]. This discrepancy can be explained by the variable roles of circadian genes in different tumors, suggesting the complexity of circadian-associated tumor development. Meanwhile, the nucleus-translocation of PER2 is noticeably suppressed in OSCC tissues and cells. The shift of PER2 from the cytoplasm to the nucleus is a normal process for the execution of its functions [22]. Thus, we speculated that loss of ability to locate into the nucleus may lead to the loss of antitumor function of PER2. Several studies investigated the reason for failure in nucleus-translocation of PER2, including the downregulation of CRY2 in caners [23]. The upstream regulator for PER2 downregulation and loss of nucleus-translocation has not reached an agreement. Up till now, PML has been shown to have a close relationship with PER2 expression and nucleus-translocation, especially for its K487 acetylation to be associated with PER2 nucleus-distribution. Also, PER2 could interact with *TP53*, which is thought to influence its time-dependent subcellular redistribution [10]. On the other hand, *TP53*, which is a well-known tumor-suppressing gene with great resistant effect on OSCC, has also been identified under the influence of PER2 expression and nucleus-translocation. This could partly explain the tumor-resistant role of PER2. It has to be admitted that the mutation of *TP53* indeed takes part in the progress of OSCC carcinogenesis. But when it comes to other groups of OSCC patients without a mutation in *TP53*, loss of PER2 might account for their downregulation of *TP53* during tumorigenesis. Collectively, the loss of the antitumor function of PER2 is closely associated with its downregulation in expression and nuclear translocation.

Our study also exhibited that the expression pattern of PER2 is aberrant in ANT with its nuclear translocation being suppressed, similar to that in OSCC. Adjacent noncancerous tissues possess the potential to undergo a malignant transformation process to obtain cancerous features while its biological behavior still maintained normal. The inflammatory tumor microenvironment is an indispensable participant in tumor metastasis, the key step of which is the release of tumor-associated cytokines [24]. Therefore, we speculated that changes occurring in ANT might be ascribed to influence cytokines from the tumor microenvironment.

In OSCC, PER2 deficiency results in a decrease of *TP53* and an increase of EMT-related genes. *TP53* is a well-known antitumor protein involved in repairing and eliminating damaged cells [25]. *TP53* deficiency plays central roles in the development of various cancers [26]. Some studies have focused on the interaction of *TP53* and PER2. PER2 is directly regulated by *TP53* binding to a response element in its promoter [27]. PER2 also leads to the increased stability of P53 by blocking ubiquitination and transcription of P53 target genes [28]. Thus, the resistance of PER2 to carcinogenesis can be partly ascribed to the activation of downstream antitumor signals of *TP53*. EMT is essential for the process of tumor metastasis [29]. Here, we showed that loss of PER2 enhanced tumor invasion by activating transcriptional genes of EMT, including *TWIST1/2*, *ZEB1/2,* and *MMP1*, suggesting a wide-range influence of PER2 on EMT process.

## 5. Conclusion

Our results demonstrated that PER2 plays an important role in tumorigenesis and the development of OSCC. Thus, our study provided strong evidences that the aberrant expression and subcellular localization of PER2 may be used as a novel biomarker for human OSCC.

## Figures and Tables

**Table 1:** The clinical characteristics of subjects.

	OSCC (*n* = 36)	ANT (*n* = 36)	NT (*n* = 36)	*P* value
Gender, *n* (%)				
Male	20 (55.6)	20 (55.6)	19 (52.8)	*P* > 0.05
Female	16 (44.4)	16 (44.4)	17 (47.2)	
Age (years)^1^	51.3 (8.2)	51.3 (8.2)	48.5 (10.9)	*P* > 0.05
Staging, *n* (%)				
T1/2	26 (72.2)	26 (72.2)		
T3/4	10 (27.8)	10 (27.8)		
Lymph node status, *n* (%)				
Positive	6 (16.7)	6 (16.7)		
Negative	30 (83.3)	30 (83.3)		
Metastasis status, *n* (%)				
Positive	0 (0.0)	0 (0.0)		
Negative	36 (100.0)	36 (100.0)		
Differentiation, *n* (%)				
Well	13 (36.1)	13 (36.1)		
Moderate	17 (47.2)	17 (47.2)		
Poor	6 (16.7)	6 (16.7)		

OSCC, oral squamous cell carcinoma tissues; ANT, adjacent noncancerous tissues; NT, normal oral tissues. ^1^Data are means (SD).

## Data Availability

The data used to support the findings of this study are available from the corresponding author upon request.
